# Sensory impairment reduces money sharing in the Dictator Game regardless of the recipient’s sensory status

**DOI:** 10.1371/journal.pone.0230637

**Published:** 2020-03-23

**Authors:** Anna Oleszkiewicz, Teresa Kupczyk

**Affiliations:** 1 Institute of Psychology, University of Wroclaw, Wroclaw, Poland; 2 Dept. of Otorhinolaryngology, Smell and Taste Clinic, TU Dresden, Germany; 3 General Tadeusz Kosciuszko Military University of Land Forces Wroclaw, Wroclaw, Poland; Universidad Loyola Andalucia Cordoba, SPAIN

## Abstract

Altruism varies as a function of minimal social cues. Sensory impaired individuals elicit more altruistic behaviors, at the same time being more prone to be exploited. We tested whether information about recipient’s sensory impairment (blindness or deafness or no impairment) would increase of the amount of money given to the anonymous partner in the Dictator Game (DG). We manipulated information about sensory status of a fictional recipient by indicating their sensory impairment (the same as the participant) or not. Sample of DG players included blind (n = 99) and deaf (n = 74) individuals and their fully functional counterparts (n = 197). Age, socioeconomic status (SES), and education were controlled. We observed higher offers in the sighted and hearing subjects as compared to sensory impaired subjects, regardless of information about the recipient’s sensory status.

## Introduction

The indirect examples of altruistic behavior towards handicapped or impaired individuals are dated far back in the history of human kind. Fossils of individuals with severe locomotive, visual and hearing impairments indicate that despite these disabilities, they lived up to 40 years, speculatively due to the acts of support from other members of the group [1–6]. Supporting handicapped, who are not likely to fully reciprocate raised a notion that altruism among ancient hominins is more similar to ours in contrast that of non-human primates. Altruistic acts in chimpanzees have been acknowledged [7–9], but their impairment is acquired in adolescence or adulthood, thus they are integrated into the group. The acts of altruism conditioning survival of young, congenitally impaired non-human primates seem to be absent [1] and therefore they are not likely to live up adolescence and adulthood.

Classical economic games with the most simple version—the Dictator Game (DG) are the most popular methods for measuring prosocial behaviors [10]. Originally proposed by Kahneman [11], DG became popular over the last decades, mostly because of its simplicity and accuracy in turning assumptions into measurable decisions [10] and design aimed to measure individual motive and intentions [12]. The main conclusion raised by the researchers using DG was that people are more prone to share with others at own cost (i.e. perform altruistic acts) than *homo economicus* theory would assume–they share more often and higher amount of goods than the null transfers predicted by the rational and self-centered social exchange theorem [13]. Altruistic behavior has been well documented in economic games between strangers [12,14–18]. Generosity in the DG has been found to merely depend on the giver’s characteristics, for example the BMI [19] but rather varies as a function of even minimal social cues about the recipient [20,21]. Social distance between the giver and the recipient can enhance generosity–the more giver knows and has in common with the recipient, the more generous their offers become [22,23]. Generosity in the DG may also be enhanced by replacing anonymous recipient with a reputable charity institution [24] or the welfare of the recipient, presenting the desire to help [25]. Information about health and cognitive abilities of the recipient can also have a significant impact on altruistic behavior. For instance, blindness of the recipient elicits more helping behavior [26] but this effect might depend on the anticipated communication possibilities. In studies involving deaf-blind individuals as help recipients, possessing a communication card predicted effective solicitation of assistance [27,28]. Prosocial behavior towards sensory impaired groups also varies as a function of engagement in the community service aimed to help these groups. In a recent study, students who got engaged in Deaf community service during their practical course of service learning, reported higher altruistic attitudes towards Deaf community as a result of this intervention [29].

There are 1.3 billion people (17.7% of world’s population) with sight abnormalities, of whom 36 millions are completely blind [30]. The population of people with disabling hearing abnormalities is estimated to 360 million people (5.3% of the world’s population). Altogether, partial or complete visual and auditory impairments might affect a significant part of the population. This large fraction of the population is however understudied and their economic behaviors have been poorly understood. It remains unclear, whether altruistic behavior exceeding the framework of *homo economicus* paradigm can be applied to specific groups of sensory impaired individuals. Existing knowledge allows to assume that blind and deaf people would receive more goods shared in the DG, but how they behave being set in a role of the Dictator remains unclear. To this end, we performed an experimental study, where we asked blind, deaf and fully functional subjects to play the DG. Within the DG we manipulated information about the sensory status of the recipient (a fictional person). The aim of the current investigation was to explore generosity of blind and deaf individuals towards other people in general as well as the role of solidarity centered on the sensory impairment in enhancing altruistic behavior.

## Materials and methods

### Ethics statement

The study was performed in accordance to the Declaration of Helsinki on Biomedical Studies Involving Human Subjects. Written informed consent was obtained from all of the subjects. The study design and consent approach were approved by the Institutional Ethics Review Board, the Polish Association of the Blind and the Polish Association of the Deaf.

### Participants

We determined the sample size by utilizing G*Power software [31]. Within the linear multiple regression with three predictors and three covariates (described in details in the Statistical analyses section), to obtain power of .90 with α = .05 to detect a small-to-medium effect of *f* = .05, the projected sample size was at least 355 subjects. The study was carried out on a sample of 370 people. The participants were recruited through the Polish Association of the Blind and the Polish Association of the Deaf and by means of personal contacts of the participants established in the course of previous projects carried at the University. The sighted participants were additionally recruited through press and social media releases and leaflets. All subjects received a monetary remuneration for their participation in this study.

### Deaf subjects

Of 103 hearing-impaired subjects invited to participate in this study we included 74 profoundly deaf individuals. All subjects who were included in the sample declared profound deafness–their hearing threshold for both ears exceeded 90dB which means practical useless of the hearing ability in social interactions. Thirty-one subjects had no hearing aid, 10 used cochlear implant, 32 used hearing aid and 1 subject did not provide this information. Seventy subjects were categorized as early-deaf (lost hearing ability before age 6 years, considered to be critical age threshold for language acquisition) and four were categorized as late-deaf (auditory loss onset later than 6 years of age). Onset of auditory loss was independent from the type of hearing aid, χ2(2) = 5.19, *p* = .08. Majority of the subjects were raised by both hearing parents (n = 57) and only some of them by both deaf parents (n = 13) or one deaf parent (n = 1); missing information (n = 3). To further assure profound deafness in the social context we performed auditory testing comprising vocal audiometry and the triplet test. Subjects performed the test while wearing hearing aid or cochlear implant. For audiometric testing we used web-based software (e-audiologia.pl) [32,33] and Sennheiser HD 280 Professional headphones. Vocal audiometry is a procedure aimed to test subject’s ability to understand common and simple words. Subject hears a word in the headphones and is required to indicate it out of the five distractors, i.e. words of different meaning, displayed on the computer screen. In the triplet test subjects were required to repeat a span of three digits they heard in headphones under conditions of varying sound-to-noise ratio. After hearing the stimulus, subjects were asked to repeat the digit span using digital keyboard displayed on the computer screen. All subjects qualified for the study did not exceed 50% of understanding of words in vocal audiometry in any of their ears regardless of the decibels Hearing Level (dbHL) and only two reached 50% of understanding in the triplet test at the sound-to-noise ratio of -6 and -10.5 points. Control sample consisted of one hundred hearing people aged between 16 and 57 years and was matched in terms of age, *t*(172) = .22, *p* = .83 and sex, χ2(1) = .15, *p* = .70. For details see Table 1.

**Table 1:** Descriptive statistics of the age and sex distribution in the blind subsample.

			Age			
	n	% females	Min	Max	M	SD
Deaf	74	50	16	55	30.74	11.46
Hearing	100	53	16	57	31.13	10.1

### Blind subjects

Ninety-nine blind subjects were recruited. Of those were 51 were congenitally blind adults and 48 late blind adults (i.e., individuals who lost their vision after age 2). Among late blind adults, sight loss duration ranged from 0.5 to 52 years (*M* = 19.1 ± 13.4). Control sample included 97 sighted people aged between 17 and 57 years and was slightly imbalanced in terms of age, *t*(194) = .-3.39, *p* = . < .001 wherein control subjects were on average 4.9 years younger than blind individuals, however the samples were balanced in terms of sex, χ2(1) = .73, *p* = .39. For details see Table 2.

**Table 2:** Descriptive statistics of the age and sex distribution in the blind subsample.

			Age			
	n	% females	Min	Max	M	SD
Blind	99	46.5	17	57	36.5	10
Congenitally blind	51	43	17	57	32.7	8.7
Late-blind	48	50	20	57	40.5	9.8
Sighted	97	52.6	17	57	31.6	10.1

### Procedure

Data were collected during individual sessions with each participant that lasted approximately 20 min and was held in a dedicated laboratory. Deaf subjects who could not read instructions or experimenter’s lips, were accompanied by a professional sign language translator. Blind subjects were assisted with trained research assistants who carefully read instructions to them. At the beginning an experimenter presented each participant with a consent form and briefly explained the protocol. We measured behavioral altruism and readiness to share with others by the means of Dictator Game utilizing 10x1PLN coins (1PLN = approximately 0.3USD). In the Dictator Game subjects received 10 coins from the experimenter and were asked to arbitrary share it with an anonymous person sitting in the next room. When giving the instructions, we manipulated information about the sensory status of the recipient–they either shared sensory disability with the sensory impaired subject (blindness in the case of blind individuals and deafness in the case of deaf individuals) or they had fully functional sight and audition. Recipient was a fictional character. Information about the fictional recipient in our study was randomized at the time when instruction was provided to the subjects. There were two versions of instruction indicating: (1) that the recipient is blind (in the case of blind-sighted subsample) or deaf (in the case of deaf-hearing subsample); (2) that the recipient is fully functional. The version of instruction was randomly drawn (for the exact instruction see S1 Appendix). The participants were debriefed immediately after the decision about money transfer was made. We explained the idea of the experiment and very briefly presented the goal of the study to the subjects. Subjects were asked to return the money they left for themselves and together with the money they transferred it was deposed with the research assistant for the next experimental sessions. Subjects were informed that this money is included in their participation remuneration. We controlled for socioeconomic status (SES) and education that both may affect decisions in the economic games [34,35]. We measured SES with a direct question *Which of the statements best describes financial situation of your household*: *1-poor*, *we cannot fulfill the basic needs*, *5 –very good*, *we can afford luxury*. Education was quantified as a number of school years one has accomplished. Age of the subjects was also controlled due to the variation in visual and auditory impairments prevalence with age. All experimental conditions were between-subjects.

### Statistical analyses

Statistical analyses were performed with Jamovi 1.0.2 software. Comparison between deaf and control subjects was performed with independent-sample t-tests and showed that SES and education were imbalanced in favor for the control sample. In blind individuals we observed lower SES than in the control sample but the number of accomplished school years between these two groups was similar (see Table 3). Therefore, we included age, SES and education as predictors in the regression models. To investigate the effects of blindness and deafness of the giver and the recipient on the amount of money shared in the DG we examined two regression models. In Model 1 we included the type of sensory impairment, the sensory status of a giver and the sensory status of a recipient as predictors and age, SES and education as covariates. Further, we tested whether congenital vs late onset of sensory impairment had an effect on the amount of coins shared in the DG (Model 2) with onset of sensory impairment and the sensory status of a recipient as predictors, and age, SES and education as covariates.

**Table 3:** Comparison between SES and education in blind-sighted and deaf-hearing subsamples. Education = number of accomplished school years.

	Socioeconomic status (SES)				Education			
	M (SD)	*t*	df	p	M (SD)	*t*	df	p
Deaf	3.27 (.72)	2.46	172	0.02	12.70 (2.45)	3.57	170	< .001
Hearing	3.52 (.61)				14.11 (2.65)			
Blind	3.30 (.60)	2.58	194	0.01	15.29 (3.74)	1.13	180	0.26
Sighted	3.53 (.61)				15.85 (2.75)			

## Results

Both regression models presented satisfactory model fit explaining 3% (Model 1) and 4% (Model 2) variance in the amount of money transferred by the Dictators. There was no collinearity between predictors as the Variance Inflation Factors (VIFs) were < .99 in Model 1 and <1.04 in Model 2. We found that the sensory status of a giver was a sole significant predictor within Model 1 (B = -.95, *p* = .007) showing that sensory impaired subjects transferred approximately .95 PLN less than their controls. Model 2 additionally revealed that this effect was mainly driven by lower offers of the subjects with early onset of sensory impairment (B = -1.13, *p* = .003) than the controls (see Table 4).

**Table 4:** Regression coefficients for Model 1 testing the effects of blindness and deafness of the giver and the recipient on the amount of money shared in the DG, and for Model 2 testing the effect of onset of sensory impairment on the amount of money shared in the DG. In both models age, SES and education are included as covariates.

			95% Confidence Interval			
Predictor	B	SE	Lower	Upper	t	p
**Model 1: R**^**2**^ **= .03**							
Intercept	5.04	1.35	2.39	7.70	3.73	< .001
The type of sensory impairment (0–Blind; 1-Deaf)	-.16	.36	-.87	.55	-.44	.66
The sensory status of a giver: (0 –Control; 1—Sensory impaired)	-.95	.35	-1.64	-.27	-2.73	.007**
The sensory status of a recipient (0—Control; 1—Sensory impaired)	.13	.34	-.54	.80	.38	.70
Age	.02	.02	-.02	.05	1.02	.31
SES	< .001	.27	-.53	.53	-.01	1.00
Education	.06	.06	-.05	.17	1.07	.29
**Model 2: R**^**2**^ **= .04**							
Intercept	4.86	1.31	2.29	7.42	3.72	< .001
Onset of sensory impairment:						
early onset—controls	-1.13	.38	-1.89	-.38	-2.96	.003**
late onset—controls	-.40	.51	-1.41	.61	-.78	.44
The sensory status of a recipient (0—Control; 1—Sensory impaired)	.10	.34	-.57	.77	.30	.77
Age	.01	.02	-.02	.04	.82	.41
SES	.03	.27	-.51	.56	.09	.93
Education	.07	.05	-.04	.18	1.31	.19

**—p < .01

**Fig 1:**
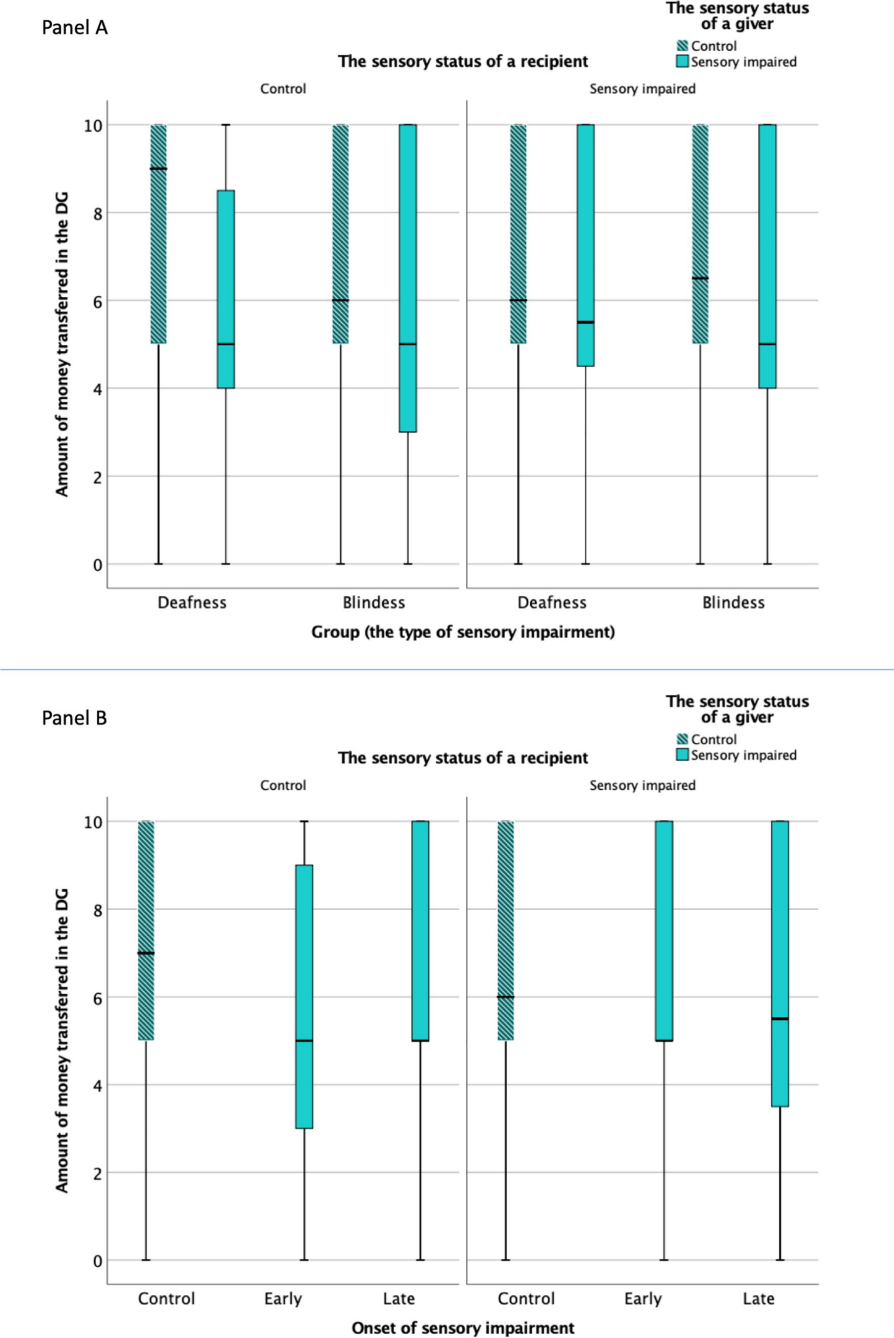
Boxplots illustrating the distribution of offers across the experimental groups. Panel A: bars (from left to right) present offers made by 1) the hearing givers to a hearing recipient, 2) the deaf giver to a hearing recipient, 3) the sighted giver to a sighted recipient, 4) the blind giver to a sighted recipient, (5) the hearing giver to a deaf recipient, 6) the deaf giver to a deaf recipient, 7) the sighted giver to a blind recipient, 8) the blind giver to a blind recipient. Panel B: bars (from left to right) present offers made by 1) the fully functional givers to a fully functional recipient, 2) the early-onset sensory impaired subjects to a fully functional recipient, 3) the late-onset sensory impaired subjects to a fully functional recipient, 4) 1) the fully functional givers to a sensory impaired recipient, 5) the early-onset sensory impaired subjects to a sensory impaired recipient, 6) the late-onset sensory impaired subjects to a sensory impaired recipient.

Fig 1 presents distribution of offers across the experimental groups. Panels in the Figure correspond with the regression models (Model 1 –Panel A; Model 2 –Panel B).

## Discussion

Sensory impairment reduces the readiness to share money at own cost, regardless of the type of sensory impairment (blindness or deafness) and sensory status of the recipient (sensory impaired or fully functional). This effect is mainly derived from the early-onset of sensory impairment, whereas losing ability to see or hear later in life does not significantly reduce behavioral altruism measured with the DG. To our best knowledge, this study is the first experimental investigation looking into the role of solidarity centered on the sensory impairment in enhancing altruistic behavior by manipulating sensory status of the recipient and including large groups of blind and deaf individuals to play the DG.

The DG measures individual motive and intentions [12] and therefore current results can be interpreted from the perspective of increased value of money given to the player with sensory impairment as compared the fully-functional player. Blind and deaf individuals were less satisfied with their household economic situation than their control sub-samples. This difference reflects the Ombudsman Office reports of a poor economic situation of the sensory-impaired groups [36]. On an individual level, one can assume that barriers stemming from their sensory impairment make it more difficult to earn money in real life and consequently money given to the sensory impaired individuals within the DG would constitute a greater value to the sensory impaired subject than their fully functional controls resulting in lower readiness to share it [37–39]. Reduced readiness to share money was independent from the type of sensory impairment of the giver (blindness or deafness), suggesting that both loss of vision and audition similarly translate into socio-economic difficulties, e.g. transition to low-paid jobs or dependency on social support system. Interestingly, we observed that the effect of sensory impairment of the giver was dependent on its onset–congenital sensory loss was associated with significantly lower transfers as compared to late onset of sensory loss. A possible explanation for difference is early development of generous economic attitude that remains practiced after the sensory loss. This could be investigated in social neuroscience paradigm by exploring brain activations during economic games in blind and deaf individuals with varying onset of sensory loss.

The amount of money transfer observed in this study was independent from the sensory status of the recipient (sensory impaired or not). This effect could be interpreted with a notion of increased selfishness of sensory impaired subjects as compared to fully-functional controls. However, mean donations in the DG (M_donation_ = 5.93 PLN) were not particularly low, in fact they exceeded half of the money players received from the experimenter. Therefore, we would rather state that sighted and hearing subjects were particularly generous (M_donation_ = 6.98 PLN). This generous economic behavior may be rooted in relatively high satisfaction with on socioeconomic status reported by the subjects and/or relatively low stakes in the DG. To further address this hypothesis a follow up study involving higher stakes would be necessary. In the light of former reports showing that even minimal social cues can alter altruistic behavior [12,14–18,20,21,23], including welfare of the recipient [25] or reputation of an institution [24], we speculate that information about sensory impairment was not immediately linked with welfare of the recipient by the givers or simply that the subjective value of money was a stronger factor in decision making process than was the sensory status of the recipient. Sensory impaired subjects in our study did not present solidarity or empathy with the anonymous recipient based solely on the information on shared disability. This is in line with former results presenting null effect of empathy manipulation on economic decisions in the DG [40]. The reasons underlying this null-result should be further studied, perhaps with qualitative methods such as in-depth interviews and could include social values orientation [41] and social motives [42]

A certain limitation of the current study refers to the difference in instructions given to the participant that was forced by their sensory impairment. Subjects with visual impairment were instructed orally by the experimenter, whilst some of the deaf subjects received written instructions and those who were not able to read were accompanied by the trained sign language translator. It is possible that the way instruction was given could influence behavioral decision, for instance through social desirability bias. Therefore, an uncontrolled demand effect [43] could have arisen that is not possible to estimate post-hoc. This should be addressed in the future studies, however the difference in instructions in studies including both blind and deaf individuals seems rather difficult to overcome. Notably, these instructions were most natural to the subjects and included their dominant way of communication. Another issue important to discuss refers to the deception of the participants. Inconsistently with the instructions given to the participants, in our study there was no actual recipient of the money transferred by the Dictators. However, DG protocol does not require to monitor reciprocal movements between the players [12] making Dictators decision complete action space of the game [10]. Further, the stakes we have used in our study were approximately 3$ which is less than usual 10$, however it has to be noted that by using the basic monetary unit in Poland (1PLN) we assured higher ecological validity of the study. By using 10x1PLN subjects could better imagine the value of money and share it according to their representation of this value. Furthermore, the value of 10 PLN in Poland is similar to the value of 10$ in the US–with these sums similar goods can be purchased on the market.

In summary, the current study presents perspective on altruistic behavior in the DG presented by non-student, large sample of blind and deaf subjects. We show that sensory impairment is associated with lower generosity that is likely to be caused by the increased subjective value of money in sensory impaired subjects. These results shed light into economic behaviors of blind and deaf individuals who constitute a large fraction of world’s population that is understudied and whose socio-economic behavior and decision making is poorly understood. Partial or complete visual and auditory impairments might affect a significant part of the population (17.7% of the global population is estimated to suffer from visual impairments; 5.3% of the global population suffers from hearing abnormalities) [44]. Although studying these large social groups of visually and auditory impaired people offers researchers a chance to better understand brain plasticity and sensory compensation mechanisms, evidence of the consequences of visual or hearing loss on social functioning and decision making remains surprisingly scarce. Reports from the field allow expecting that information about sensory impairment of the recipient would elicit more altruistic behaviors. However, it is unclear how sensory impaired people would act in a situation when they decide whether and to what extent act altruistically and whether information about sensory impairment of the recipient enhances their readiness to act altruistically. Our results showed that sensory impairment of the giver reduced offers in the DG regardless of information about the sensory status (impaired vs not) of the recipient. Therefore, the current study challenges previous statements that the minimal social cues about the recipient can change decision in economic games and suggests that own situation (in this case sensory status of the giver) might override the role of information about the recipient.

## Supporting information

S1 Appendix Game instructions. (DOCX)

S2 Data (XLSX)
