# Supplementary material for: Sensory impairment reduces money sharing in the Dictator Game regardless of the recipient’s sensory status
Source: PLoS One. 2020 Mar 23;15(3):e0230637. doi: 10.1371/journal.pone.0230637 (PMC7089535; doi:10.1371/journal.pone.0230637)
Supplement: S1 Appendix — (DOCX) [file pone.0230637.s001.docx]

Appendix 1: Game instructions

**Instructions read to blind/sighted subjects**:

In front of you there are 10 coins of 1 PLN. You can decide how many coins you wish to transfer to a **blind/sighted*** person sitting in the next room. Please indicate what amount of money you would like me to transfer to the person sitting in the next room.

*information whether a fictional partner was blind or sighted was randomized

Debriefing: In this task we intended to measure readiness to share with others. 10 PLN that you have shared will be included in your remuneration for participation in our study. We kindly ask to return us the coins for the purpose of testing next participants.

**Instructions read to deaf/hearing subjects**:

In front of you there are 10 coins of 1 PLN. You can decide how many coins you wish to transfer to a **deaf/hearing*** person sitting in the next room. Please indicate what amount of money you would like me to transfer to the person sitting in the next room.

*information whether a fictional partner was deaf or hearing was randomized

Debriefing: In this task we intended to measure readiness to share with others. 10 PLN that you have shared will be included in your remuneration for participation in our study. We kindly ask to return us the coins for the purpose of testing next participants.
